# Engineered probiotics

**DOI:** 10.1186/s12934-022-01799-0

**Published:** 2022-04-27

**Authors:** Junheng Ma, Yuhong Lyu, Xin Liu, Xu Jia, Fangyun Cui, Xiaoheng Wu, Shanshan Deng, Changwu Yue

**Affiliations:** 1 Key Laboratory of Microbial Drugs Innovation and Transformation, Medical College, Yan’an University, Yan’an, 716000 Shaanxi China; 2 Non-Coding RNA and Drug Discovery Key Laboratory of Sichuan Province, Chengdu Medical College, Chengdu, 610500 Sichuan China; 3 School of Public Health, Chengdu Medical College, Chengdu, 610500 Sichuan China; 4 School of Basic Medical Sciences, Chengdu Medical College, Chengdu, 610500 Sichuan China; 5 Ecological Environmental Monitoring Center, Luoyang, 471000 Henan China

**Keywords:** Engineered probiotics, Synthetic biology, Gene editing, Disease diagnosis and treatment, CRISPR, Genetic engineering

## Abstract

Engineered probiotics are a kind of new microorganisms produced by modifying original probiotics through gene editing. With the continuous development of tools and technology progresses, engineering renovation of probiotics are becoming more diverse and more feasible. In the past few years there have been some advances in the development of engineered probiotics that will benefit humankind. This review briefly introduces the theoretical basis of gene editing technology and focuses on some recent engineered probiotics researches, including inflammatory bowel disease, bacterial infection, tumor and metabolic diseases. It is hoped that it can provide help for the further development of genetically modified microorganisms, stimulate the potential of engineered probiotics to treat intractable diseases, and provide new ideas for the diagnosis of some diseases or some industrial production.

## Background

Probiotics have been studied for decades. The word "probiotics" first appeared in 1974, and, in 2001, the Food and Agriculture Organization of the United Nations and the World Health Organization defined probiotics as "live microorganisms which when administered in adequate amounts confer a health benefit on the host" [1, 2]. Probiotics play a great role in many aspects, such as preventing and treating various clinical diseases, improving the intestinal microenvironment, inducing immune regulation, preventing physiological stress, inhibiting the growth of pathogens, and improving the barrier function of the intestinal epithelium, etc. [3]. Probiotics are therefore of great interest to the scientific community.

In recent years, genome sequencing has become more affordable and some of the tools for editing and modifying microbial genomes have become more powerful, enable us to engineer probiotics according to our own ideas, so that we can develop customized probiotics [4]. By means of gene editing, probiotics can have a variety of beneficial properties, and can treat specific diseases, which is beneficial to human health. Genetic engineering of microbial strains such as probiotics is a promising research (Table 1). We are at a turning point in the research of probiotics. Engineered probiotics may become new ideas and new methods to solve some problems. This review briefly introduces the theoretical basis of gene editing technology and focuses on some recent engineered probiotics studies on diseases, including inflammatory bowel disease, bacterial infection, tumor and metabolic diseases. We hope to provide help for the further development of engineered probiotics.

**Table 1:** Summary of engineered probiotics for diagnosis and therapy

Disease	Probiotic used	Object	Refs.
*Clostridioides difficile* infection	An *S. boulardii* strain	Mice	[5]
Cancer	*E. coli* Nissle 1917	Mice	[6]
Cancer	*E. coli* Nissle 1917	Mice	[7]
Cancer	*E. coli* Nissle 1917	Mice	[8]
Cancer	*E. coli* Nissle 1917	Mice	[9]
Cancer	*E. coli* Nissle 1917	Mice and human	[10]
Cholera	*L. lactis* CSL	Mice	[11]
Colitis	*E. coli* Nissle 1917	Mice	[12]
Diabetes	*L. gasseri* ATCC 33,323	Rats	[13]
Hyperammonemia	*E. coli* Nissle 1917	Mice and human	[14]
Inflammatory bowel disease	*E. coli* Nissle 1917	Mice	[15]
Inflammatory bowel disease	*E. coli* NGF-1	Mice	[16]
Inflammatory bowel disease	*E. coli* Nissle 1917	In vitro	[17]
Inflammatory bowel disease	*E. coli* Nissle 1917	Mice	[18]
Inflammatory bowel disease	*E. coli* Nissle 1917	Mice	[19]
Inflammatory bowel disease	Yeast strain BS016	Mice	[20]
Inflammatory bowel disease	*E. coli* Nissle 1917	Mice	[21]
*Listeria* infection	*L. casei* ATCC334	Mice	[22]
Obesity	*E. coli* Nissle 1917	Mice	[23]
Phenylketonuria	*L. reuteri* 100-23C	Mice	[24]
Phenylketonuria	*E. coli* Nissle 1917	Human	[25]
*Pseudomonas aeruginosa* Infection	*E. coli* Nissle 1917	Caenorhabditis elegans and mice	[26]
*Staphylococcus aureus* Infection	*L. reuteri* DSM20016	In vitro	[27]
Ulcerative colitis	*E. coli* Nissle 1917	Mice	[28]
Vancomycin-resistant *Enterococci* infection	*E. coli* Nissle 1917	Mice	[29]

## Theoretical basis of engineered probiotics-gene editing

Through gene editing, the existing probiotics are modified to obtain the desired new probiotics. Such engineering modification allow us to directly verify whether the genetic material, proteins and functional roles of these novel microorganisms have been changed as desired. Gene editing technology continues to develop, from homologous recombination to the first-generation Zincfinger Nucleases (ZFNs) technology, to the second-generation Transcription Activator-like Effector Nucleases (TALEN) technology and to the more popular third-generation Clustered Regularly Spaced Short Palindromic Repeats CRISPR associated (CRISPR-Cas) technology in recent years [30]. Among them, ZFN and TALEN were selected as the method of the year by Nature Methods, CRISPR gene editing technology was selected as the best breakthrough of 2015 by Science news, and CRISPR-Cas9 technology won the 2020 Nobel Prize in Chemistry, which has a revolutionary impact on life sciences. This part will mainly give a brief introduction to these three generations of technologies.

These gene-editing tools introduce Double Strand Break (DSB) into target genes, DNA Repair is induced by an error-prone Non-homologous End Joining (NHEJ) pathway or Homology Directed Repair (HDR) [31]. ZFNs and TALEN are two artificial restriction endonucleases which use zincfinger DNA domains or TAL-effect DNA domains to edit or cut specific target DNA [32]. However, there have been some problems in the use of these two technologies, such as the lack of specificity of ZFN and easy introduction of non-targeted mutations [33]. The construction of standard ZFNs and TALENs is time-consuming and laborious [34]. Therefore, although ZFNs and TALENs have been used in gene editing of human, animal, and plant cells since 2002 and 2011, these limiting factors have hindered their wide application to a certain extent [35]. Since the end of 2012, researchers have turned their attention to the CRISPR technology, which has more powerful editing efficiency and is simpler and more flexible to use, so that the application of this technology brings a whole new direction to gene editing [36].

CRISPR and related Cas genes are considered to be part of the bacterial immune system, which is a self-protective sequence of bacteria and can provide phage resistance [37]. At the molecular level, bacteria integrate the virus gene from the first invasion into their CRISPR spacer, and when the virus invasions again, CRISPR transcribed to produce the precursor CRISPR RNA (pre-crRNA), which is further processed to contain crRNA that matches the viral gene sequence. CrRNA recognizes homologous sequences of viral genomes and mediates binding to viral genes and cleavage of Cas proteins [38, 39]. The CRISPR-Cas system is generally classified by the Cas protein gene adjacent to CRISPR. The currently reported CRISPR-Cas system is mainly divided into 2 classes, 6 types and 33 subtypes [40]. The nucleases Cas9 and Cas12a (Cpf1) in the 2 classes of Cas protein family are more widely used which are easy to edit to identify specific DNA sequences [41]. Among them, the type II CRISPR-Cas9 system is currently the more commonly used protein for genome editing [42].The CRISPR-Cas9 system is composed of Cas9 protein, crRNA and trans-activating RNA (tracrRNA). The Cas9 protein is the only protein with DNA catalytic activity among the many CaS proteins in *Streptococcus thermophilus* [43], It snips double-stranded DNA at sequence targets through crRNA matching [30]. By artificial design, crRNA and tracrRNA can be converted into small Guide RNA (sgRNA), which can guide Cas9 to perform site-specific DNA cutting, gene knockout and insertion [44].

CRISPR technology can be used not only for the study of loss of function such as CRISPR knock out (CRISPRKO) and CRISPR interference or inhibition (CRISPRi), but also for the screening study of gain of function such as CRISPR activation (CRISPRa) [30]. The development of gene editing technologies especially CRISPR provides essential supports for the emergence of the next generation of probiotics.

## Research and application of engineered probiotics

Advances in gene editing technology provide new possibilities for gene editing of probiotics. Engineered probiotics have developed rapidly in recent years. Researchers have conducted a variety of biomedical studies on engineered probiotics for disease diagnosis and treatment. In particular, several engineered probiotics have also entered the clinical trial stage (Table 2). Scientists have also developed engineered microorganisms that are relevant to industrial applications. These studies are briefly discussed below.

**Table 2:** Examples of engineering probiotics in clinical trials

Species	Engineered probiotic	Disease/function	Research facility	Stage	Result	ClinicalTrials.gov identifier
*E. coli*	SYNB1934 SYNB1618	Phenylketonuria	Synlogic	Phase 1	–	NCT04984525
*E. coli*	SYNB1618	Phenylketonuria	Synlogic	Phase 1/2a	–	NCT03516487 NCT04534842
*E. coli*	SYNB8802	Enteric Hyperoxaluria	Synlogic	Phase 1	–	NCT04629170
*E. coli*	SYNB1891	Metastatic Solid Neoplasm and Lymphoma	Synlogic	Phase 1	–	NCT04167137
*E. coli*	SYNB1020	Cirrhosis and Hyperammonemia	Synlogic	Phase 1/2	Terminated	NCT03447730
Bacteroides*	NB1000S	Enteric Hyperoxaluria	Novome	Phase 1/2a	–	NCT04909723
*Lactococcus lactis*	AG013	Oral Mucositis	Oragenics/Precigen ActoBio	Phase 2	Terminated	NCT03234465
*Lactococcus lactis*	AG019	Type 1 diabetes	Precigen ActoBio	Phase 1/2	–	NCT03751007
*B. longum*	bacTRL-IL-12	Solid Tumours	Iqvia Pty Ltd	Phase 1	–	NCT04025307

*The researchers did not list specific species

### Engineered probiotics and inflammatory bowel disease

Microbiota imbalance is closely related to the development of inflammatory bowel disease (IBD). Probiotics have been shown to improve the microbiome of the microbiota by changing the intestinal environment and inhibiting the growth of harmful bacteria, also, they can prevent inflammatory diseases from further affecting the host immune system, and have positive significance for the regulation of inflammation [45, 46]. Advances in gene editing technology have increased the link between probiotics and inflammatory bowel disease.

Specific probiotics have been designed to diagnose and treat IBD. Around 2017, researchers have used engineered bacteria to diagnose IBD [15–17]. Based on biomarkers such as thiosulfate, tetrasulfate, and nitric oxide (NO), they constructed stably genetically engineered bacteria as recognition elements of biosensors to diagnose IBD. In recent years, research on the use of engineered probiotics for the treatment of IBD has also emerged. Pichet Praveschotinunt and others of Harvard University genetically engineered *E. coli* Nissle 1917 (ECN) to produce a curly fiber matrix that promotes intestinal epithelial integrity, and the trefoil factor (TFF) component in the matrix promotes intestinal barrier function and epithelial repair [18]. Their study confirmed that the engineered ECN could in situ produce proteins that were protective against dextran sodium sulfate (DSS)-induced colitis model mice. This provides an engineered probiotic treatment for IBD. Engineered ECN is also used by many other researchers to treat intestinal-related inflammation. Researchers engineered ECN to express interleukin-10 (IL-10), ketone body (R)-3-hydroxybutyrate (3HB) and Schistosoma immunomodulatory protein Sj16 and other substances. These substances can protect the intestinal mucosa by promoting the growth of probiotics, inhibiting the growth of harmful bacteria, improving the intestinal microenvironment, and downregulating inflammatory response-related cells or proteins, so as to achieve the purpose of treating and relieving the symptoms of intestinal inflammation [12, 19, 28]. However, these studies have only made progress in mouse models. We believe that after further clinical trials, these engineered ECNs must have the opportunity to be used for the relief and treatment of human IBD. Benjamin M. Scott of the University of Maryland and Cristina Gutierrez-Vazquez of Harvard Medical School conducted a valuable study based on yeast. They developed a self-regulating engineered yeast probiotic that can express human P2Y2 purinergic receptors and they linked the activation of the probiotic’s P2Y2 receptor with the secretion of ATP-degrading enzymes, enabling it to sense pro-inflammatory molecules and neutralize pro-inflammatory molecules through self-regulation and secretion of corresponding proteins [20]. This self-regulating engineered yeast probiotic inhibits intestinal inflammation and reduces intestinal fibrosis and dysbiosis in IBD mouse models and we think their self-regulating strategy may provide a new way for engineered probiotics to treat IBD or other diseases. Liu Jinyao's team at Shanghai Jiaotong University School of Medicine in China synthesized a polydopamine nano immunosuppressant and used it to coat *E. coli* Nissle 1917 to inhibit the excessive immune response in the local tissues of mouse colitis and regulate the intestinal microbiota to promote the reversal of inflammation [21]. Liu et al.'s study showed that applying immunosuppressants on the surface of beneficial bacteria can promote improved colitis responses in mice. Through their research, can we engineer probiotics to express immunosuppressants to wrap themselves to treat inflammatory disease?

### Engineered probiotics and bacterial infections

Bacterial infections have a great impact on people’s health and nearly one million people die from bacterial infections worldwide each year, and the lack of bacterial resistance and new antibiotics has become an obstacle to solving the problem of bacterial infections [47]. Researchers have found some new ways to alleviate this problem.

Some researchers have successfully developed engineered probiotics. The American team of Arun K. Bhunia designed an engineered *Lactobacillus casei* strain that can produce *Listeria* adhesion protein [22]. This strain is colonized in the intestine of mice, competitively reducing the colonization of *Listeria* mucosa and systemic transmission, protecting mice from fatal infections. They can also enhance the intestinal immune regulation function by accumulating intestinal mucosal regulatory T cells, CD11c^+^ dendritic cells and natural killer cells. The method of engineering *L. casei* ATCC334 to reduce *Listeria* infection and protect the intestinal tract is worth learning. Some researchers have designed and constructed an engineered probiotic based on *E. coli* Nissle 1917, which can specifically target and kill the two most common vancomycin-resistant *Enterococcus*, and significantly reduce the number of *Enterococcus faecalis* and *Enterococcus faecium* in the feces of model mice [29]. Some researchers have engineered *Saccharomyces boulardii* so that the engineered bacteria secrete a fusion protein (ABAB) that can neutralize 4 different *Clostridium difficile* toxins [5]. The preventive administration of these bacteria can significantly relieve the inflammation and tissue damage related to the infection of *Clostridium difficile* in the mouse intestines mucosa, therefore reduce the mortality of these mice. In addition, researchers have developed some engineered probiotics to better prevent and prevent and diagnose bacterial infections. Some researchers have genetically modified *Lactobacillus lactis* to produce engineered probiotics that recognize the cholera autoinducer 1 (CAI-1) produced by *Vibrio cholerae* and cause color changes in feces [11]. Another researcher has constructed an engineered *lactic acid bacteria* that can be used to detect real-time changes in autoinducer peptide-I (AIP-I) produced by *Staphylococcus aureus* [27]*.* The researchers also engineered *E. coli* Nissle 1917 to specifically kill pathogens by detecting the autoinducer N-acyl homoserine lactone (AHL) of *Pseudomonas aeruginosa* and releasing antibiotics and anti-biofilm enzymes [26]. This series of studies may provide a new direction for the treatment and prevention of drug-resistant bacterial infections. It is hoped that the safety and effectiveness of these engineered probiotics can be further confirmed, and these products can serve the clinic as soon as possible.

### Engineered probiotics and tumors

The discussion about the treatment of cancer by bacteria has existed for decades [48, 49]. Some probiotics can induce anti-cancer effects by enhancing the apoptosis of cancer cells and preventing oxidative stress [50–52]. With the development of modern gene editing technology, it has become possible to design probiotics that can play a positive role in fighting cancer. Researchers are constantly searching and developing new probiotics to fight tumors.

In the study of Candice R. Gurbatri and others at Columbia University, researchers used synthetic biology methods to modify bacteria and obtained an engineered *E. coli* strain subtype called "SLIC" [6]. SLIC colonizes tumor cells, when it proliferates to a certain extent, it can spontaneously lyse and then release PD-L1 and CTLA-4 nano antibodies. So that SLIC can promote T cell activation, increase memory T cells, and enhance anti-tumor immune response, thereby effectively inhibiting tumor growth, causing tumor regression, and inhibiting tumor metastasis. Their research has positive significance for enhancing the effect of tumor treatment and inhibiting tumor cell proliferation. In a similar study, researchers in China engineered *E. coli* Nissle 1917 to target the angiogenic inhibitor TUM-5 and tumor suppressor p53 to the anaerobic tumor region [7]. This treatment significantly inhibited the growth of transplanted tumor in mice, and their idea of using the tumor tropism of some bacteria to target tumors provides a direction for the use of anti-tumor drugs. An engineered *E. coli* Nissle 1917 that can be targeted to colonize tumors has also been published recently. It can convert the metabolic waste produced by tumors into L-arginine that enhances the anti-tumor immune response, and can effectively enhance the therapeutic effect of programmed cell death protein-1 and its ligand inhibitors on mouse tumors [8]. An engineered *E. coli* Nissle 1917 that can achieve precise tumor treatment in vivo through the control of light color changes has also been successfully constructed^(9)^. These studies have provided some help for the treatment of tumors. Now there are two engineering probiotics related to the treatment of tumors in clinical trials (NCT04167137 and NCT04025307). The most notable of these is the SYNB1891 strain obtained by the engineering of *E. coli* Nissle 1917 by Synlogic (NCT04167137). This strain can produce cyclic di-AMP to stimulate the interferon gene pathway, and then trigger innate immunity by activating antigen-presenting cells to present tumor antigens [10]. Their phase1 clinical trials are underway, and we believe that the emergence of this engineered probiotic drug will be seen clinically in the near future.

### Engineered probiotics and metabolic diseases

The content of metabolism-related substances is generally in a relatively stable state in the body. When the biochemical process in the body is hindered, the accumulation or lack of certain metabolites can cause diseases [53–55]. These diseases are difficult to treat, and often require long-term control of living and eating habits, which brings serious economic burdens and life pressures to patients [51]. Engineered probiotics can break down or transform these accumulated substances to help treat such diseases.

Phenylketonuria (PKU) is caused by mutations in the phenylalanine hydroxylase gene. Patients with phenylalanine hydroxylase deficiency cannot effectively decompose phenylalanine, causing phenylalanine to accumulate in the blood, which seriously affects the patients’ health [53]. The researchers expressed the phenylalanine lyase gene in *Lactobacillus reuteri* 100-23C, so that the engineered probiotic strain can produce phenylalanine lyase, which can reduce the blood phenylalanine of the PKU mouse model [24]. Their experiment provides an idea for the treatment of phenylketonuria with engineered probiotics. However, the strain may colonize the host body for a long time, which is also a problem waiting to be solved. Synlogic also conducted a similar study. They inserted the genes encoding phenylalanine ammonia lyase and l-amino acid deaminase into the genome of *E. coli* Nissle 1917, and constructed a strain of *E. coli* named SYNB1618 [25]. This engineered probiotic strain can consume phenylalanine in the gastrointestinal tract and will not colonize the intestinal tract. It has clinical safety (NCT03516487). And they also extended the model used to predict the therapeutic potential of SYNB1618 based on this study, which combined in vitro experiments and knowledge of human physiology to predict the degree of plasma phenylalanine reduction by SYNB1618 [56]. This facilitates the use of the biologic SYNB1618. Follow-up clinical trials for SYNB1618 are also underway (NCT04534842 and NCT04984525), and it is hoped that this bioactive drug can bring good news to patients with phenylketonuria.

Mutations in the alanine glyoxylate aminotransferase gene can cause hyperoxalic acid. The accumulation of glyoxylic acid can lead to the increase of oxalate and even the production of urinary stones [54]. Synlogic has also constructed an orally engineered *E. coli* strain called SYNB8802, which can ingest oxalic acid in the gastrointestinal tract to reduce urinary oxalic acid levels and reduce kidney damage caused by hyperoxaluria. Results from a Phase 1a study of this trial demonstrated the strain's safety profile, and the later trials are still in progress (NCT04629170). Novome has also developed a drug named NOV-001 for the treatment of intestinal hyperoxalic acid. This drug is composed of engineered strain NB1000S and plant-derived polysaccharide NB2000P. It is now undergoing clinical trials to verify its efficacy (NCT04909723). In our opinion, engineered probiotics could be a good way to treat diseases in combination with other drugs.

Ammonia is a highly neurotoxic metabolite. Impaired ammonia clearance occurs when urea cycle enzyme deficiency or liver cell dysfunction occur. If hyperammonemia is not treated in time, coma or even death may occur [55]. Researchers genetically modified the oral probiotic *E. coli* Nissle1917 to obtain an engineered strain named SYNB1020. This strain can convert ammonia into L-arginine, reduce the ammonia content in the intestine of a mouse model of hyperammonemia, and improve the survival rate of mice [14]. However, in patients with cirrhosis, SYNB1020 was discontinued due to lack of efficacy (plasma ammonia AUC) compared to placebo (NCT03447730). Clinical trials in patients with urea cycle disorders are still underway (NCT03179878), hoping to produce a good result.

Researchers have also developed engineered probiotics for some other metabolic-related diseases. Some researchers have constructed an engineered commensal *Lactobacillus gasseri* that secretes glucagon-like peptide-1 (GLP-1) to improve diabetes [13]. GLP-1 secreted by this strain can reprogram intestinal cells into insulin-producing cells, thereby reducing hyperglycemia in mice. Similar studies have shown an engineered EcN-GM that induces GLP-1 expression, which may have a beneficial effect on obesity, hyperglycemia and liver steatosis [23]. Precigen ActoBio has genetically modified *Lactococcus lactis* to obtain a engineered bacteria named AG019. AG019 can deliver proinsulin and interleukin-10 to the gastrointestinal mucosa tissues, reduce or eliminate the damage to pancreatic β cells, and potentially stabilize or improve endogenous insulin production. Their experiment Also in progress (NCT03751007).

### Engineered bacterias and industrial production

Engineered probiotics can not only be used for the diagnosis and treatment of some diseases, but also be closely related to industrial production. Jaewoo Son and others of the Korea Academy of Science and Technology used the CRISPRi system to develop an engineered *Leuconostoc citrate* [57]. The strain down-regulated the expression of two genes and introduced a co-expression operon to increase the production of riboflavin. Recently, researchers conducted similar studies on *E. coli* LS31T to increase the production of riboflavin [58]. Thus the industrial production of riboflavin, as a food additive, may find a new direction. Photosynthetic cyanobacteria convert carbon dioxide into monosaccharides, and some researchers have designed an engineered *E. coli* that can further convert the products of photosynthetic cyanobacteria into 2,3-butanediol [59]. The study of this engineered microbe is worth digging deeper, and perhaps in the future it can really provide propellant for rocket launches and be used as return material for interstellar travel, just as researchers designed. Studies have reported an engineered *E. coli* strain that can produce benzoic acid from plant-derived glucose [60]. Their research may provide a safer new idea for the production of benzoic acid as a preservative. Some researchers engineered *Clostridium ljungdahlii* to produce isopropanol, 3-hydroxybutyric acid, ethanol and other substances [61], which research provides conditions for the use of single-carbon source materials to produce a large number of chemicals. There are also some studies providing a platform for the production of some industrial substances such as L-citrulline, flavan-3-ols, β-carotene, etc. by designing *E. coli*, *lactic acid bacteria* and yeast into engineered bacteria [62–66].

### Other research on engineered bacterias

Zbiotics has added an acetaldehyde dehydrogenase gene that decomposes acetaldehyde in *Bacillus subtilis*, which can further convert the acetaldehyde converted from ethanol into acetic acid to reduce the harm of alcohol. They used the spores of this strain to conduct a 90-day repeated administration toxicological evaluation on mice, and proved that the bacterium has no side effects [67]. Zbiotics is the first company in the world to produce and sell genetically engineered probiotic products. Although their products are already on sale, the safety of this bacteria in the human body may still need to be demonstrated. To use CRISPR for genetic modification of microorganisms. Some researchers have constructed engineered *Lactobacillus plantarum* WCFS1 to produce n-acetylglucosamine [68]. Some researchers have found that the modified *Brucella* ATCC MYA-796 can ferment galactose faster [69, 70]. There are also more and more researchers constructing gene editing platforms for engineered bacteria such as *Corynebacterium glutamicum*, *Candida parapsilosis*, *Lactobacillus casei*, *Lactobacillus reuteri*, *Bifidobacterium*, *Clostridium butyricum*, and *Escherichia coli* [71–78]. They successfully applied CRISPR-related technology to the genetic modification of microorganisms. Their research will certainly provide help for the application of this technology in engineered probiotics, and the engineering of probiotics based on this technology will surely make it easier to produce the desired engineered probiotics.

## Prospects

The emerging gene-editing methods used to develop synthetic engineered probiotics have great potential for the treatment and diagnosis of different types of human diseases, and engineered probiotics may become a new method for the treatment of cancer, inflammation, infection and some other diseases. The engineered probiotics designed by the researchers are more effective, have fewer side effects and are more affordable than traditional treatments or wild-type strains, benefiting more patients and families. The advantages of engineering probiotics such as stability, specificity, preference, low cost, and relative safety may make them a new choice for the treatment of many of the diseases mentioned above and more. However, the application of engineered probiotics still faces a series of challenges. For example, many microorganisms have few genetic tools that can be manipulated, and the microorganisms currently used for research design are only a small part of the vast family of microorganisms, which leads to the restriction of probiotic modification to a few strains. There are still a few engineered probiotics that go from the laboratory to the clinic, and even if they enter clinical trials, it cannot be guaranteed that it can achieve the intended effect, which hinders the efforts of some researchers to construct engineered probiotics. Some argue that genetically modified organisms (GMOs) are harmful, preventing the effective deployment of engineered probiotic therapies.

## Conclusions

Despite these limitations in the research and application of engineered probiotics, we cannot ignore their potential role in improving human health. Success and failure are inevitable in the research and development of engineered probiotics. We need more research to find the possibility of success, not only in the research of various probiotic modification tools, but also in-depth understanding of disease pathology, so as to make it easier for us to design the desired probiotics. The efficacy of engineered probiotics is a problem that we must consider, but before these probiotics undergo clinical transformation, safety issues must be resolved. It is necessary to ensure that the guidelines established by various health regulatory agencies are followed to design and construct safe and effective probiotics. We believe that with the increasing understanding of the human microbiome and disease mechanisms, the rapid development of gene editing technology, safe, stable and effective engineered probiotics will surely shine in many fields.

## Data Availability

Not available.
