# Correction to: Engineered probiotics

**DOI:** 10.1186/s12934-022-01820-6

**Published:** 2022-05-24

**Authors:** Junheng Ma, Yuhong Lyu, Xin Liu, Xu Jia, Fangyun Cui, Xiaoheng Wu, Shanshan Deng, Changwu Yue

**Affiliations:** 1 Key Laboratory of Microbial Drugs Innovation and Transformation, Medical College, Yan’an University, Yan’an, 716000 Shaanxi China; 2 Non‑Coding RNA and Drug Discovery Key Laboratory of Sichuan Province, Chengdu Medical College, Chengdu, 610500 Sichuan China; 3 School of Public Health, Chengdu Medical College, Chengdu, 610500 Sichuan China; 4 School of Basic Medical Sciences, Chengdu Medical College, Chengdu, 610500 Sichuan China; 5 Ecological Environmental Monitoring Center, Luoyang, 471000 Henan China

## Correction to: Microbial Cell Factories (2022) 21:72 https://doi.org/10.1186/s12934-022-01799-0

In the original version of the article [1], Xin Liu was incorrectly denoted as being one of the equally contributing authors in the author group though it was not mentioned in the footnote. The symbol indicating the equally contributing author was removed from Xin Liu in the author group.

The original article has been corrected.
